# Adaptive HIV pre-exposure prophylaxis adherence interventions for young South African women: Study protocol for a sequential multiple assignment randomized trial

**DOI:** 10.1371/journal.pone.0266665

**Published:** 2022-04-13

**Authors:** Jennifer Velloza, Nicole Poovan, Nontokozo Ndlovu, Nomhle Khoza, Jennifer F. Morton, Jeanne Omony, Edwin Mkwanazi, Cole Grabow, Deborah Donnell, Richard Munthali, Jared M. Baeten, Sybil Hosek, Connie Celum, Sinead Delany-Moretlwe

**Affiliations:** 1 Department of Global Health, University of Washington, Seattle, WA, United States of America; 2 Wits Reproductive Health & HIV Institute (Wits RHI), Faculty of Health Sciences, University of The Witwatersrand, Johannesburg, South Africa; 3 Vaccine and Infectious Disease Division, Fred Hutchinson Cancer Research Center, Seattle, WA, United States of America; 4 Department of Epidemiology, University of Washington, Seattle, WA, United States of America; 5 Department of Medicine, University of Washington, Seattle, WA, United States of America; 6 Department of Psychiatry, Stroger Hospital of Cook County, Chicago, IL, United States of America; New York Blood Center, UNITED STATES

## Abstract

**Introduction:**

Pre-exposure prophylaxis (PrEP) is a highly effective HIV prevention strategy and is recommended for populations at risk of HIV, including adolescent girls and young women (AGYW) in HIV endemic settings. However, PrEP continuation and high adherence remain challenges to its impact. Existing PrEP adherence interventions can be time- and cost-intensive. Widescale PrEP delivery will require the identification of layered PrEP support strategies for AGYW with diverse prevention needs. We describe the design of a sequential multiple assignment randomized trial (SMART) to evaluate a PrEP adherence support model using scalable, stepped interventions in AGYW in South Africa.

**Methods:**

“PrEP SMART” is a randomized trial in Johannesburg, South Africa, enrolling AGYW who are between 18 and 25 years of age, sexually active, newly initiating PrEP, and have regular access to a mobile phone. Participants are randomized 1:1 to standard-of-care PrEP counseling with either two-way SMS or WhatsApp group adherence support. Adherence is assessed at three months using tenofovir diphosphate (TFV-DP) levels from dried blood spots collected at month 2 to categorize participants as “responders” (TFV-DP ≥500 fmol/punch) or “non-responders” (TFV-DP <500 fmol/punch). AGYW defined as ‘non-responders’ undergo a secondary 1:1 randomization to either quarterly drug-level feedback counseling or monthly issue-focused counseling, in addition to their first-level intervention. The primary outcome is PrEP adherence at nine months (TFV-DP ≥700 fmol/punch). We will assess the effect of our two initial interventions on TFV-DP levels among responders, assess the effect of our intensified interventions on TFV-DP levels among non-responders, and identify the optimal sequence of adherence interventions through nine months.

**Trial registration:**

ClinicalTrials.gov, NCT04038060. Registered on 30 July 2019.

## Introduction

Adolescent girls and young women (AGYW) between the ages of 18–25 are a high risk population for HIV acquisition and recent HIV prevention trials have reported HIV incidence rates of approximately 4 per 100 person-years in this population, in the context of risk reduction counseling, treatment of sexually transmitted infections, and condom provision [1–3]. In sub-Saharan Africa specifically, it is estimated that 50% of new HIV infections occur among young women under the age of 25 years [4]. HIV pre-exposure prophylaxis (PrEP) is a core biomedical intervention for populations at risk of HIV acquisition including AGYW [5, 6]. However, while randomized controlled trials conducted with men who have sex with men and HIV serodiscordant couples found high PrEP efficacy (i.e., 42%-74% efficacy and >90% effectiveness among persons with drug levels indicating high adherence), studies with AGYW have reported low PrEP adherence and continuation for this younger population [7–14]. The World Health Organization recommends oral PrEP for AGYW and PrEP has been approved for HIV prevention in over 100 countries [15]. It is being scaled up globally, including in South Africa, a country with the world’s highest HIV incidence among AGYW. The South African Department of Health recently launched an initiative to roll out PrEP in over 1000 primary health care clinics nationwide [16, 17]. PrEP uptake has generally been high in countries where it is being delivered, and South Africa currently has approximately 100,000 PrEP users of 213,990 target users [16, 18]. In 2019, 13 of the country’s 17 PrEP demonstration and implementation projects (76.5%) were specifically targeted toward AGYW [18].

Efficacy trials of tenofovir (TFV)-emtricitabine (FTC) PrEP demonstrated that adherence is a key factor in the level of protection that oral PrEP provides against HIV infection [19, 20]. However, despite promising PrEP initiation rates (with 95–100% of those screened and eligible for PrEP initiating PrEP), PrEP adherence and continuation during periods of HIV risk remain a challenge for some AGYW with retention rates around 50% by Month 12 and 15% by Month 24 [21]. In PrEP demonstration projects and delivery programs with AGYW, PrEP continuation rates declined to around 18–55% over the first six months and adherence was approximately 30% by 12 months [9, 13, 14, 21–23], due to individual, interpersonal, and contextual factors, including lack of support, difficulty with habit formation, side effects, stigma related to PrEP and HIV, gender-based violence (GBV), and depression and stress [14, 24–27]. Visit frequency and contact with providers may also be a factor; PrEP demonstration projects which explored the importance of visit frequency with young people found that PrEP adherence among South African AGYW generally declines as clinic visits move from monthly to quarterly [9, 11, 13, 23, 28]. It is necessary to understand and address barriers to PrEP adherence and continuation among AGYW to achieve greater public health benefits of PrEP.

Several PrEP adherence support interventions have been tested among adolescent populations at risk of HIV, including mHealth strategies (e.g., one-way and two-way SMS messages, smartphone applications), in-person adherence clubs, psychosocial counseling, and tailored feedback based on the amount of PrEP detected in pharmacologic samples or electronic PrEP adherence data [9, 22, 29–31]. However, these approaches have either been evaluated as stand-alone interventions or as part of a package. Therefore, results from these PrEP studies do not provide evidence about the optimal sequence of combination adherence interventions that is most effective for AGYW. These interventions can be quite cost- and time-intensive, and widescale PrEP delivery will require understanding the characteristics of AGYW who adhere well to PrEP with only minimal adherence support and identifying layered intensive strategies that will work for those who need more intensive support. A stepped PrEP adherence support model has the potential to be feasible, resource-saving, and similar to differentiated care models for HIV in which frequent visits with more intensive support are directed to the minority of patients who may benefit from them most [32, 33].

## Materials and methods

### Trial objectives and outcomes

**Fig 1:**
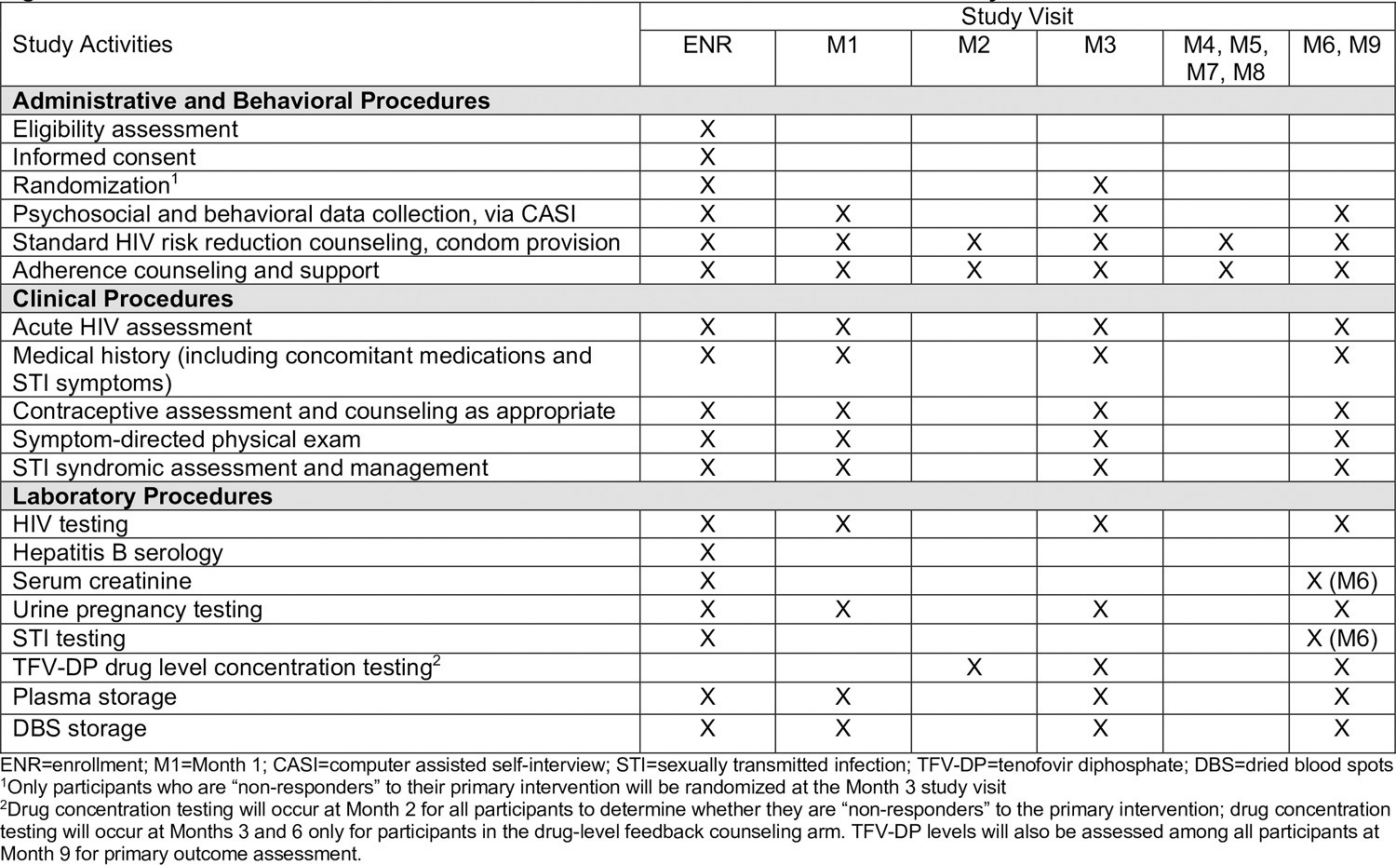
Schedule of enrollment, interventions, and assessments for PrEP SMART study. ENR = enrollment; M1 = Month 1; CASI = computer assisted self-interview; STI = sexually transmitted infection; TFV-DP = tenofovir diphosphate; DBS = dried blood spots. ^1^Only participants who are “non-responders” to their primary intervention will be randomized at the Month 3 study visit. ^2^Drug concentration testing will occur at Month 2 for all participants to determine whether they are “non-responders” to the primary intervention; drug concentration testing will occur at Months 3 and 6 only for participants in the drug-level feedback counseling arm. TFV-DP levels will also be assessed among all participants at Month 9 for primary outcome assessment.

**Fig 2:**
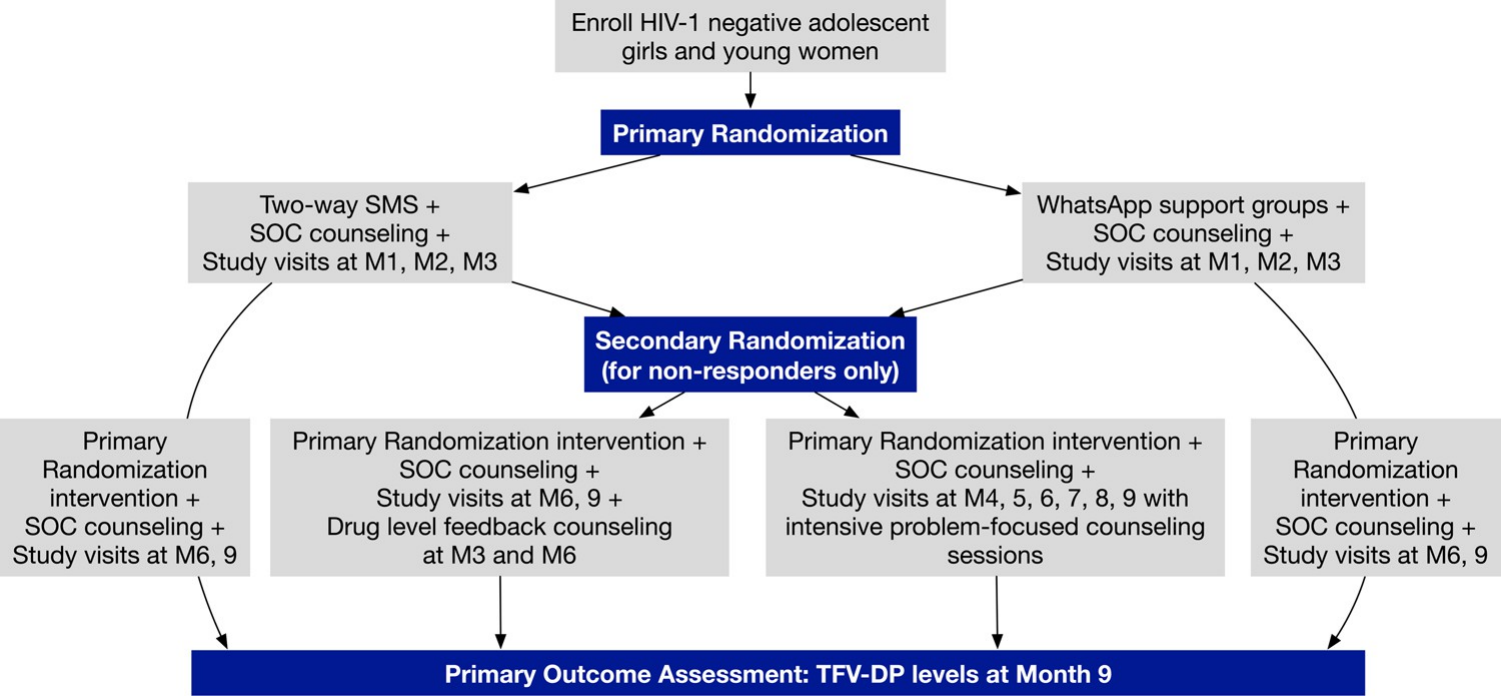
Flow diagram of study enrollment, randomization, and intervention arms. ^1^A participant is considered a “non-responder” if they have Month 2 TFV-DP levels <500 fmol/punch or missed PrEP refills at the Month 1 and/or Month 2 study visits. Secondary randomization takes place at the Month 3 study visit.

The primary goal of the present study is to test a stepped model of scalable adherence support strategies for South African young women who initiate PrEP, using a single-site prospective sequential multiple assignment randomized trial (SMART) design (ClinicalTrials.gov identifier: NCT04038060; Standard Protocol Items–Recommendations for Interventional Trials [SPIRIT] checklist in **S1 File**). We aim to enroll up to 500 AGYW to ensure that we retain approximately 350 AGYW who complete at least three months of study procedures in Johannesburg, South Africa. This will ensure that we follow AGYW and provide PrEP interventions to the group interested in PrEP after the first several months of use, when PrEP drop-off is expected to be high. We will follow participants for nine months to assess the interventions’ effect on PrEP adherence (**Figs 1 and 2**). Trial conduct and reporting will be informed by the Consolidated Standard of Reporting Trials (CONSORT) evidence-based recommendations for randomized trials.

#### Objectives

This trial (referred to as “PrEP SMART”) has three specific objectives. First, we aim to evaluate the proportion of young South African women who adhere well to PrEP with mHealth interventions (i.e., two-way SMS, WhatsApp support groups) through two months of PrEP use and compare PrEP adherence between the two mHealth arms. Second, we aim to evaluate, among those who do not achieve PrEP adherence by two months, the proportion who adhere well to PrEP after receiving one of the two intensified interventions (monthly visits with issue-focused counseling, quarterly visits with counseling based on PrEP blood levels, called drug-level feedback counseling). This analysis will be restricted to those with low PrEP adherence in the first two months, defined as having tenofovir diphosphate (TFV-DP) drug concentration less than 500 fmol/punch or missing PrEP refills. Additional details are provided in the “Rationale for SMART Design” section below. Third, we will evaluate the optimal sequence of intensifying adherence support.

#### Outcomes

The primary outcome is PrEP adherence, measured via DBS TFV-DP drug concentration ≥700 fmol/punch at nine months. TFV-DP provides a marker of cumulative PrEP dosing in the prior month. The threshold of 700 fmol/punch was associated with high efficacy in open-label use of PrEP and with 4–5 PrEP doses per week in directly observed dosing studies [34, 35]. In the iPrEx study among men who have sex with men (MSM) and transgender women (TGW), the TFV-DP threshold of 700 fmol/punch represented dosing ≥ 4 times a week and was associated with 100% efficacy [36]. A similar threshold of TFV-DP and dosing frequency associated with efficacy among cisgender women has not been established.

Secondary outcomes include: PrEP adherence, analyzed as continuous TFV-DP levels as well as in a five-category variable (below the limit of quantification [BLQ], BLQ–349 fmol/punch, 350–699 fmol/punch, 700–1249 fmol/punch, and ≥1250 fmol/punch). We will also explore the shorter-term impact of our interventions on TFV-DP levels at two months.

### Rationale for SMART design

Adaptive SMART designs have been previously used to address questions of stepped interventions for other health issues (e.g., mental health treatment) [37, 38], but the application of this design to HIV prevention is relatively novel. SMARTs differ from other randomized trials by allowing for the identification of “responders” and “non-responders” to initial interventions, the optimal sequence of interventions, and the comparison of individual characteristics between persons who respond to the intervention sequences [37, 39, 40].

At enrollment, PrEP SMART participants are randomly assigned to receive standard PrEP adherence counseling with *either* participation in weekly two-way SMS or WhatsApp adherence support groups. At Month 3, young women who have low PrEP adherence (based on TFV-DP <500 fmol/punch from a DBS sample at their Month 2 visit) or those who have missed PrEP refills (at Month 1 or Month 2 study visits), undergo a secondary randomization to either quarterly visits with drug-level feedback counseling at Month 3 and Month 6 visits *or* monthly follow-up visits with issue-focused counseling through Month 9, while continuing with their primary intervention. While the TFV-DP threshold of 700 fmol/punch will be used for our primary outcome assessment as previously described, we will use a threshold of 500 fmol/punch at the two-month study visit to determine whether a participant is a “responder” or “non-responder,” which corresponds to an average of 2–3 doses per week [36]. It is a more sensitive threshold to classify consistent dosing after only two months of PrEP use and will ensure that we are allocating additional adherence support resources to participants with the greatest difficulty taking PrEP regularly.

### Rationale for study interventions

Two-way SMS messages were informed by behavioral models highlighting strengthened communications between patients and providers as a way to improve ART adherence among Kenyan adults living with HIV [41]. The WelTel intervention demonstrated the efficacy of weekly two-way text messages asking how patients were doing, with telephone support for participants who responded that they were not doing well [42]. A recent meta-analysis found that two-way text messaging can be effective for antiretroviral therapy (ART) adherence, particularly with messaging provided on a less than daily basis and tailored content and timing [43]. The recent HPTN 082 study built off this evidence base to develop a two-way SMS intervention for PrEP adherence among AGYW in South Africa and Zimbabwe [29]. Study participants were sent a weekly discrete SMS message, “Are you well, girlfriend?”, and were asked to respond “yes” or “no”, where “no” responses could indicate that they needed a staff member to follow up and discuss side effects, adherence challenges, or other issues. Participants who responded “no” or those who did not respond received a call-back from staff within 24 hours. This intervention was feasible and acceptable for AGYW and PrEP SMART follows a similar model.

WhatsApp groups provide a virtual platform for peers to chat and offer each other social and emotional support [44]. In PrEP SMART, WhatsApp groups are meant to serve as a virtual peer support platform in lieu of in-person adherence support clubs. Prior research has found that peer support clubs improve adherence among outpatients starting ART [44–46], hard to reach patients [47], and adolescents [48]. Peer groups may improve adherence by offering social and emotional support to encourage the management of health behavior [49]. Evidence from recent PrEP studies (HPTN 082, EMPOWER) has shown that monthly, in-person peer adherence clubs were well-attended and highly acceptable among AGYW initiating PrEP [26, 50]. In HPTN 082, 50% of participants attended three or more peer support/adherence clubs in the first six months of PrEP use. However, in-person groups posed logistical barriers to participation, particularly for AGYW with competing school and work priorities, and are difficult to implement in the context of regional lockdowns and requirements for social distancing during the COVID-19 pandemic. WhatsApp groups may overcome barriers to in-person adherence groups while impacting health behavior change through peer support and coping skills. The use of WhatsApp also leverages the uptake of social media by many youth worldwide.

The rationale for the use of drug-level feedback counseling first arose from an early PrEP randomized trial where women reported a desire for feedback on their PrEP adherence through pharmacologic testing and directed counseling [27]. More recently, HPTN 082 tested a PrEP adherence support package that included drug-level feedback counseling based on TFV-DP levels in DBS obtained at the prior visit. This study did not find statistically significant differences in adherence levels between the retrospective drug-level feedback counseling and standard counseling arms during follow-up [29]. However, notably, 31% of women in the drug-level feedback counseling arm did not receive their counseling because DBS results were not available at the visit, as they had to be batch-shipped and performed, or they did not receive the appropriate counseling message due to miscoding of the laboratory results [29]. Retrospective drug-level feedback also may not have improved PrEP adherence because it was provided one month after the DBS sample was collected, and reflected adherence in the four-to-six weeks prior to the visit at which the DBS sample was taken. It may have been difficult for women to remember their pill-taking behavior and adherence barriers from approximately two months before they received the counseling. In addition, HPTN 082 provided drug-level feedback counseling alongside monthly PrEP adherence support clubs and two-way SMS messages, and had an observed HIV incidence of 1.0 per 100 person-years, which was significantly lower than the estimated HIV incidence of 3.7% from a counterfactual estimate of HIV incidence, indicating the success of this intervention package overall and making it difficult to disentangle the effect of only drug-level feedback on PrEP adherence [51]. We will be able to improve the drug-level feedback counseling procedures using key lessons learned in HPTN 082 and, through our SMART design, we have the unique potential to answer questions about the impact of drug-level feedback for AGYW in this stepped-up care approach.

Monthly counseling visits were informed by prior open-label PrEP studies in the US (ATN 110 and 113) and South Africa (PlusPills) which found a significant drop in PrEP adherence among adolescents when visit intervals increase from monthly to quarterly [11, 13, 23, 28]. We hypothesize that youth who have difficulty adhering to PrEP after the first three months of follow-up will continue to need more frequent contact and specialized adherence counseling when establishing adherence habits.

### Study setting

The study is taking place at the Wits RHI Ward 21 Clinical Research Site (CRS), affiliated with the University of the Witwatersrand, Johannesburg, South Africa. The CRS is located in a Provincial Department of Health care facility, “Ward 21.” Ward 21 is one of the largest HIV clinics in the country, with a PEPFAR-supported youth clinic that provides comprehensive care for young people between the ages of 10–24 years.

### Study oversight and ethical review

Prior to initiating study activities, participants are allowed sufficient time to review study information, discuss with staff and make an informed decision about participation. Specifically, potential participants receive the informed consent form when they enter the clinic and are then told to take their time reviewing and to let staff know when they are finished. A PrEP SMART staff member then reviews the consent form with them and answers any participant questions, before conducting a comprehension assessment where the participants are asked a series of questions about information in the informed consent form. They must answer all questions correctly before they can sign the consent form and are given two attempts to complete the comprehension assessment. On average, this process takes about one hour to complete. Informed consent is being documented in accordance with the Declaration of Helsinki. All recruitment, study, and data collection procedures have been approved by ethics review boards at the University of Washington and the University of the Witwatersrand (initial protocol version 1.0 was approved on 14 January 2019; protocol version 2.0 with modifications to remote visit procedures due to COVID was approved on 26 August 2020). The study has an external Data Monitoring Committee (DMC), comprised of experts in HIV prevention, mobile phone interventions, and PrEP delivery among young women. The DMC meets every six months to review study progress, adverse events, HIV seroconversions, and interim data (in closed sessions) and provides recommendations to the investigator team.

### Patient and public involvement

PrEP SMART was developed after two-way SMS, in-person adherence support groups, and drug-level feedback counseling were found to be acceptable and feasible in HPTN 082 conducted at the same site in Johannesburg [23, 30]. Study staff, several of whom were involved in the HPTN 082 study, provided input on the design, recruitment materials, informed consent and randomization procedures, and data collection and analysis plans. A local Youth Community Advisory Board also gave feedback on study materials and we will continue to engage this group as we analyze data and disseminate findings. The study team meets bi-weekly to discuss progress, share any challenges, and problem-solve approaches to address these issues. We will seek feedback from participants, project staff, and key stakeholders via qualitative interviews during the study.

### Recruitment

Participants are being recruited from the Gauteng province of South Africa. The Ward 21 CRS team has established successful recruitment strategies, including recruitment from primary health clinics, community events, and participant referral. Recruitment is led by an experienced team of community health workers who are known and trusted in the areas where we are recruiting PrEP SMART participants. We developed a PrEP SMART recruitment flipchart to explain study rationale, procedures, and potential risks and benefits of participation using adolescent-friendly language and graphics. In response to the COVID-19 pandemic, schools were closed and individuals’ movement between geographic regions has been restricted. As a result, the team has devised new strategies to identify eligible AGYW, including recruitment via housing associations, religious groups, daycare centers, social media advertisements, and geographic and social media mapping to identify clusters of AGYW where we could target informational sessions and outreach. We also developed social media flyers to circulate via Facebook and our study clinician has held Facebook virtual sessions to answer AGYW questions about PrEP and the study. We will purposively sample potential participants to include young women between ages 18–25 years and to ensure a mix of young women who are in university and those who have less than university education.

### Study procedures

An overview of study procedures and activities is summarized in **Fig 1**. Staff were trained in all study procedures by the study investigators (CC, SDM), sub-investigator (NP), site coordinator (NN), and project director (JV). Counselors have been trained by JV and SH in counseling procedures and manuals, and JV will regularly meet with counselors and other site staff throughout follow-up to discuss intervention delivery and ensure that study procedures occurred with fidelity. Refresher trainings will be conducted approximately annually. All staff will review and sign standard operating procedures around study procedures prior to beginning their role on PrEP SMART.

#### Enrollment

Eligibility is assessed by trained staff prior to enrollment and following written informed consent (**Table 1**). All participants must be AGYW between 18 and 25 years of age and willing to initiate PrEP. At enrollment, clinical evaluations and procedures include contraception and HIV counseling and collection of a detailed medical history that includes assessment for acute HIV, pregnancy, and sexually transmitted infection (STI) symptoms. An STI symptom-directed physical exam is also performed if indicated. A participant is considered enrolled when all enrollment visit procedures are complete and she has been randomized to an mHealth intervention arm. Only de-identified data will be collected and stored to protect participant’s confidentiality and a unique study identifier will be used to link data from a participant during trial participation.

**Table 1:** Study inclusion and exclusion criteria.

Inclusion Criteria	Exclusion Criteria
• Female at birth • Age 18–25 years • Per participant report, sexually active, defined as having vaginal or anal intercourse at least once in the month prior to screening • Literate in one or more of the study languages • Willing and able to provide informed consent • Able and willing to provide adequate locator information • Regular access to a mobile phone with SMS and WhatsApp capacity • Agrees not to participate in other research studies involving drugs or medical devices for the duration of the study participation.	• Planning to relocate in the next year • Has a job or other obligations that would require long absences from the area (>4 weeks at a time) for the year • A reactive or positive HIV test at Enrollment • Any reported PrEP use within the last six months • Concomitant participation in a clinical trial using investigational agents, including placebo-controlled clinical trials using such agents • Prior participation in the active arm, or current participation in any arm, of an HIV vaccine trial • Positive pregnancy test

SMS = short message service; PrEP = pre-exposure prophylaxis

#### Randomizations

At enrollment, participants are individually randomized, in a 1:1 ratio, to either participation in a WhatsApp group or weekly two-way SMS communication. The study randomization list was created using variable-sized blocks. Specifically, the data manager (CG) created 100 blocks. Each block was assigned a random value between 0 and 1, and these values were used to assign each block with a block size (10, 12, or 14). Each observation within the block was then assigned a second random number. Assignments of “WhatsApp” were given to those with random numbers <0.50 and assignments of “2-way SMS” were given to those with random numbers ≥0.50. Random numbers were generated using the SAS “ranuni” function. Arm assignments were then placed in opaque envelopes labeled numerically (e.g., “Envelope 1”, “Envelope 2”).

The study sub-investigator is responsible for conducting randomizations, by assigning enrolled participants with study ID numbers and sequentially opening the randomization envelopes in order of increasing numeric value. The sub-investigator is an appropriate choice for conducting randomization because she is not responsible for conducting PrEP counseling, delivering study interventions, or collecting participant data. Secondary randomization at Month 3 for ‘non-responders’ follows similar procedures, with randomizations conducted in a 1:1 ratio to either monthly visits with issue-focused counseling or quarterly visits with drug-level feedback counseling. Secondary randomization assignments are stratified by primary intervention arm.

The study principal investigators are blinded to Month 9 TFV-DP outcome data by study arm (primary and secondary random assignments) for the duration of participant enrollment and follow-up.

#### PrEP provision

PrEP is offered at enrollment and follow-up visits as part of a comprehensive package of sexual and reproductive care that includes HIV testing, standard-of-care PrEP adherence and HIV counseling, condoms, contraception, and etiologic testing and treatment of curable STIs (chlamydia, gonorrhea, syphilis, trichomoniasis). Participants may decline PrEP refills at any time.

PrEP may be temporarily paused on participant request or if clinically indicated for participant safety concerns at any time. Serious adverse events (SAEs) deemed to be related to PrEP will result in a temporary hold of PrEP, which may be reinitiated once the event has resolved. PrEP will also be halted temporarily if creatinine clearance (CrCl) <60mL/min. CrCL testing will be repeated within two weeks and PrEP may be re-started if CrCL ≥60 mL/min. For the purposes of this study, only SAEs, CrCl <60 ml/min, PrEP tolerability or side effects that lead to discontinuation, and social harms associated with participation will be documented and reported to the DMC. Staff report information regarding SAEs to their institutional review board (IRB) in accordance with requirements. Participants will be referred for further physical or mental health care during trial participation as needed.

#### Primary interventions: Two-way SMS

SMS messages asking whether participants are well are sent out to participants in the two-way SMS arm on Mondays afternoons. Participants who respond “No” will receive a phone call from staff within 24 hours of their response. Participants who do not respond within 24 hours will automatically receive second message asking whether they are well. Those who do not respond to either message will receive follow-up phone calls, within 24 hours after their 48-hour window to respond to the first message has closed. Opt-out reminder messages are sent out to participants at enrollment and after the first 13 weeks of follow-up.

#### Primary interventions: WhatsApp groups

WhatsApp groups will include 25–50 participants each. We aim to have approximately 3–4 groups running throughout the study. AGYW are assigned to WhatsApp groups in batches (e.g., the first 10 participants are enrolled in Group 1, the next 10 are enrolled in Group 2), to ensure sufficient numbers for group communication early in recruitment. At enrollment, AGYW randomized to WhatsApp are entered into one of the study WhatsApp groups and the group facilitator virtually introduces her. She also receives instructions on group rules and norms. Each group is led by a staff facilitator and a young woman who is experienced in taking PrEP (a “PrEP Ambassador”) who moderate and answer questions about PrEP, facilitate discussions, introduce topics of interest, monitor member conduct and enforce group rules. Each month, facilitators develop a plan that consists of activities and topics such as “Ladies Night”, “Ask a Doctor”, games, memes, etc., to stimulate discussions. The topics are informed by participants, public holidays, and issues trending in social media and the news. The group facilitator is responsible for adding and removing individuals from the group when they exit the study, request to be removed, or violate the group rules. Groups are also given the option to meet in person, for about one to two hours approximately quarterly, to stimulate group cohesion.

#### Secondary interventions: Drug-level feedback counseling

**Fig 3:**
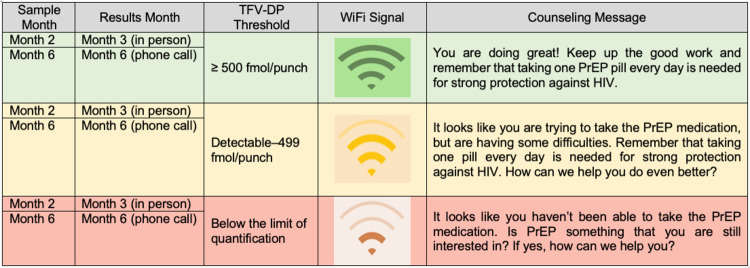
Drug-level feedback counseling messages and corresponding TFV-DP thresholds. “Sample month” refers to the month when the TFV-DP sample was collected. “Results month” refers to the month when the results were given to the participant (e.g., for blood samples drawn at the Month 2 visit, TFV-DP results were reported to participants during their Month 3 visit. For TFV-DP levels greater than or equal to 500 fmol/punch, participants were shown the green wifi signal as a visual representation of their drug levels and they were told the accompanying counseling message by the counselor. For TFV-DP levels between detectable (>16 fmol/punch) and 499 fmol/punch, participants were shown the yellow wifi signal and were told the accompanying counseling message. Finally, for TFV-DP levels below the limit of quantification, participants were shown the red wifi signal and were told the accompanying counseling message.

Women who are randomized to drug-level feedback counseling at Month 3 will move to quarterly visits and receive counseling based on DBS TFV-DP levels obtained at Months 2 and 6. Adherence counseling based on these drug levels will be provided in person at the Month 3 visit (based on results from the DBS sample taken at Month 2) and telephonically after the Month 6 visit (based on results from the DBS sample taken at Month 6). The thresholds and counseling about adherence based on drug-level feedback are shown in **Fig 3**. Study clinicians and sub-investigators will be responsible for providing drug-level feedback counseling and will be trained to use the image shown in **Fig 3** to deliver the correct “Wi-Fi” signal visual, counseling message, and follow-on discussion based on a participant’s TFV-DP DBS results. Clinical staff conducted the drug-level feedback counseling because they are more accustomed to reviewing laboratory results than lay counselors, based on our prior experience with the HPTN 082 study [23, 29].

#### Secondary interventions: Monthly issue-focused adherence counseling

Women who are randomized to monthly issue-focused adherence counseling at Month 3 will be on a monthly visit schedule and receive counseling based on PrEP adherence and other topics of the participant’s choice. At the beginning of each monthly session, counselors review facilitators and barriers to PrEP use and other salient topics in participants’ lives (e.g., relationship break-up, school exams). Counselors then complete a checklist of topics mentioned and ask the participant to choose one or two they would like to cover during the session. Counselors lead the participants through a series of interactive activities and discussion using manuals on relevant topics adapted from other HIV studies with adolescents (e.g., HPTN 082, MTN 034/REACH, ATN 110 and 113, EMPOWER). PrEP SMART counseling manuals include content on intimate partner violence, relationship issues, mental health issues such as depression and anxiety, and drug and alcohol use, as well as other topics less related to HIV prevention, including career and financial planning, school applications, social media, and internet safety. Each session lasts between 20–60 minutes. Participants are asked to provide verbal consent to record sessions for purposes of monitoring counseling fidelity.

### Data collection

Quantitative and laboratory data will be collected from all participants and qualitative data will be collected from a subset of participants. **Fig 1** provides a detailed description of which data are collected at each visit.

#### Quantitative data collection

Participants complete computer-assisted self-interviewing (CASI) surveys to assess demographic, behavioral, and psychosocial characteristics including HIV and pregnancy risk perception, depressive symptoms, intimate partner violence, HIV stigma, self-efficacy to use PrEP, self-esteem, relationship power dynamics, sexual behavior, alcohol and drug use, and PrEP readiness.

In addition, study staff collect additional data on PrEP refills and discontinuations, visit retention, pregnancy and pregnancy outcome status, medical history, laboratory results, and social harms and reportable adverse events. These data are reported on case report forms (CRFs). Staff also complete CRFs to document responses that require follow-up contact and non-responses to SMS messages among participants in the two-way SMS arm, including information on the date and outcome of follow-up phone calls to reach participants. SMS and WhatsApp conversation data are collected and de-identified through the SMS and WhatsApp platforms and key data (e.g., frequency of messages overall and by participant) are displayed on a data dashboard. CRF and CASI data will be collected and managed in REDCap and the study team will run monthly quality control reports to assess data quality and resolve queries.

#### Laboratory data

Laboratory data includes HIV testing (at enrollment, Month 1, and quarterly visits thereafter), hepatitis B serology (at enrollment), serum creatinine (at enrollment and Month 6), urine pregnancy testing (at enrollment, Month 1, and quarterly visits thereafter), and STI testing for chlamydia, gonorrhea, trichomoniasis, and syphilis (at enrollment, Month 6, and study exit). Plasma and DBS samples are collected for PrEP drug concentration testing at enrollment, Month 1, Month 2, and quarterly visits thereafter. We will use the DBS samples collected at Month 2 for our re-randomization determination and those collected at Month 9 for our primary outcome assessment. All plasma samples and DBS collected at other visits will be stored for later testing.

#### Qualitative data collection

In-depth serial interviews will be conducted with a purposive sample of 25 women. The sub-sample includes 1) young women who accept and adhere to PrEP based on PrEP drug levels at two months and 2) young women who discontinue PrEP and/or have low drug levels at two months. The sample comprises participants from each randomization arm.

These interviews occur at three visits. The first interview is after the Month 2 visit and explores the decision to take PrEP, barriers and facilitators to PrEP adherence, and experiences with the SMS messages or WhatsApp groups. The second is after their Month 6 visit and further explores barriers and facilitators to PrEP adherence and participant’s reactions to the second randomized intervention, as applicable. The third interview is conducted after the study exit visit. In this interview, participants are asked to provide a narrative history and timeline of their sexual relationships, living conditions, and other important factors in their lives. Local researchers/ethnographers are conducting all qualitative interviews with oversight from the protocol team and a social scientist. All interviews are audio-recorded and transcribed and translated into English by trained staff.

We will review qualitative data from WhatsApp and SMS messages to summarize topics discussed around barriers and facilitators to PrEP use and acceptability of study interventions.

### Statistical methods

#### Sample size and statistical power

We estimate that enrolling up to 500 participants (with approximately 350 retained through their Month 9 study visit) will provide 96% power to detect an approximately 20% difference in the proportion of participants with TFV-DP levels ≥700 fmol/punch between the two-way SMS and WhatsApp group arms at the Month 9 study visit. With 350 participants at the Month 9 study visit, we would have 84% power to detect a 15% difference in TFV-DP levels between the two mHealth interventions. Assuming that between 70–105 participants in each primary intervention arm are re-randomized at the Month 3 visit, we will have 79–96% power in each primary intervention arm to detect a 30% difference in TFV-DP levels at the Month 9 visit in the monthly counseling arm compared with the drug-level feedback counseling arm.

#### Quantitative data analysis

This study has three primary quantitative objectives, described below.

Objective 1 is to evaluate the proportion of young South African women who adhere well to PrEP with regular clinic visits and mHealth interventions. This analysis will include all enrolled participants. We will conduct an intention-to-treat analysis, using robust standard errors, to assess the effect of primary randomized intervention on Month 9 PrEP adherence (using a threshold of 700 fmol/punch) across participants in the two-way SMS arm versus WhatsApp groups. The effect of the randomized intervention will be estimated as a relative risk, with study arm assigned at the first randomization as the predictor.

Objective 2 is to evaluate the proportion of young South African women who initially did not achieve high adherence based on TFV-DP levels at month 2 and subsequently adhere well to PrEP with intensified interventions. This analysis will include participants who are re-randomized to either monthly issue-focused counseling or drug-level feedback counseling. We will assess the effect of each secondary intervention on Month 9 PrEP adherence by comparing TFV-DP levels across participants receiving monthly counseling versus drug-level feedback, conditioned on the first randomization. The effect of the randomized intervention will be estimated as a relative risk, with study arm assigned at the second randomization as the predictor. Primary randomization assignment will also be included as a covariate in the models to provide main effects of the second-stage interventions for non-responders, conditioned on the first-stage interventions.

Objective 3 is to identify the optimal sequence of adherence support among young women, where support is intensified in those who have low adherence after two months of use. This analysis will again include all enrolled participants. We will estimate the proportion with high PrEP adherence for each of the four adaptive adherence support strategies and will compare the proportion with high PrEP adherence across the four groups (**Table 2**). We will use inverse probability weights to account for non-response rates and re-randomization.

**Table 2:** Adaptive adherence support strategies.

Adaptive intervention	Decision rule
**Non-response definition:** TFV-DP levels <500 fmol/punch or missed refills prior to the Month 3 study visit	
First offer monthly SOC counseling and WhatsApp group intervention; then add drug-level feedback with quarterly visits for non-responders and switch responders to quarterly SOC visits and continued WhatsApp groups	First-stage intervention option = {SOC + WhatsApp} IF evaluation = {non-response}, THEN second-stage intervention option = {DLFB + first-stage intervention option + quarterly study visits}. ELSE continue on first-stage intervention option with quarterly study visits
First, offer monthly SOC counseling and two-way SMS intervention; then add drug-level feedback with quarterly visits for non-responders and switch responders to quarterly SOC visits and continued two-way SMS	First-stage intervention option = {SOC + SMS} IF evaluation = {non-response}, THEN second-stage intervention option = {DLFB + first-stage intervention option + quarterly study visits}. ELSE continue on first-stage intervention option with quarterly study visits
First, offer monthly SOC counseling and WhatsApp group intervention; then add monthly follow-up visits with issue-focused adherence counseling for non-responders and switch responders to quarterly SOC visits and continued WhatsApp groups	First-stage intervention option = {SOC + WhatsApp} IF evaluation = {non-response}, THEN second-stage intervention option = {Monthly visits and specialized counseling + first-stage intervention option}. ELSE continue on first-stage intervention option with quarterly visits
First, offer monthly SOC counseling and two-way SMS intervention; then add monthly follow-up visits with issue-focused adherence counseling for non-responders and switch responders to quarterly SOC visits and continued two-way SMS	First-stage intervention option = {SOC + SMS} IF evaluation = {non-response}, THEN second-stage intervention option = {Monthly visits and specialized counseling + first-stage intervention option}. ELSE continue on first-stage intervention option with quarterly visits

In addition to these three primary intention-to-treat analyses, we will also conduct modified intention-to-treat analyses only including participants who attend at least one follow-up study visit by Month 3 as a way to understand the impact of our interventions on PrEP adherence among participants with continued interest in PrEP. We will also conduct per-protocol analyses of participants who successfully received SMS, WhatsApp, monthly counseling, and drug-level feedback interventions and will explore whether the per-protocol analyses lead to different findings than the intention-to-treat analyses. Additional secondary analyses include exploring the proportion of women who achieve high PrEP adherence at Month 2, identifying sociodemographic and behavioral correlates of PrEP adherence at Months 2 and 9, assessing timing of and factors associated with PrEP discontinuation, and characterizing their level of engagement in the four interventions and intervention acceptability.

#### Qualitative data analysis

Interviews will be transcribed verbatim and translated into English. Interview data will be analyzed using thematic-content analysis and will focus on participants’ accounts of the four interventions and PrEP adherence. NVivo 12 will be used to organize and analyze data. We will follow standard approaches to thematic analysis, including memo-writing to gain familiarity with the data, generating initial codes, searching for and reviewing themes, and defining and naming themes. We will also analyze SMS and WhatsApp data using a coding and memo-writing approach.

### Impact of COVID-19 on trial progress

On 5 March 2020 the first COVID-19 case was reported in South Africa. A national lockdown was instituted on 26 March 2020. Due to lockdown regulations and guidance from ethics review boards, we temporarily stopped enrolling participants. A protocol amendment was subsequently developed and approved to allow for remote visits (with phone-based counseling and HIV and pregnancy self-testing) when appropriate. Recruitment activities resumed on 1 June 2020, while adhering to local guidance on personal protective equipment (PPE) and physical distancing. We are conducting remote visits for monthly counseling, HIV and pregnancy self-testing, and qualitative interviews via telephone when feasible, and collect quantitative and qualitative data on satisfaction with and acceptability of remote visit procedures.

### Trial status

The first participant was enrolled and randomized on 30 May 2019. We have enrolled 360 participants as of 29 January 2021 (protocol version 2.0; approval date: 26 August 2020). We expect participant follow-up and data collection to conclude in January 2022.

## Discussion

This innovative trial will assess the proportion and characteristics of AGYW adhering well to PrEP with minimal adherence support and more intensive adherence support interventions to evaluate a package of stepped PrEP adherence support interventions for AGYW at risk of HIV in South Africa. Our initial intervention approaches are “light touch” mHealth approaches which could be implemented in PrEP programmes—two-way SMS messages and WhatsApp peer support groups—followed by either monthly counseling or quarterly drug-level feedback counseling for participants who require additional adherence support. A stepped approach to adherence counseling may maximize the individual and public health impact of PrEP among young African women by supporting women who are motivated to use PrEP but encounter issues with daily pill-taking in a scalable, feasible, and resource-saving manner [6, 22]. Our study has several potential limitations. Our sample size calculations for the second objective assessing differences in PrEP adherence between monthly counseling and drug-level feedback counseling is based on hypotheses around how many individuals will be non-adherent to PrEP after the first few months in the study. However, we may have fewer re-randomized individuals and would be less able to detect a statistically significant difference in PrEP adherence between our secondary interventions. We selected a time point of three months for re-randomization (based on month 2 drug levels) given prior evidence about drop-offs in PrEP adherence and persistence among young women around this time, but our results may be different with a longer time period until re-randomization. Finally, we did not include a run-in period and may therefore re-randomize young women who wanted to try PrEP for a brief period but decided that it was not the right HIV prevention option for them.

Identifying differentiated and youth-friendly models of adherence support, tailored to AGYW’s needs, has proven successful for HIV treatment support and is particularly critical in resource-constrained settings as PrEP delivery is rolled out in already-overburdened healthcare systems [33, 52–54]. The results of this trial are expected to inform PrEP delivery strategies aimed at improving PrEP pill-taking behavior adherence and efficiency of PrEP implementation for AGYW in South Africa and other sub-Saharan African contexts. They may also be relevant to longer-acting biomedical HIV prevention formulations (e.g., cabotegravir injections, dapivirine ring) to promote ongoing engagement and persistence with these methods.

## Ethics and dissemination

Ethics approval for this trial has been obtained from the Human Research Ethics Committee at the University of the Witwatersrand in Johannesburg, South Africa (FWA #00000715) and the University of Washington’s Institutional Review Board in Seattle, Washington, USA (FWA #00006878). Written informed consent will be obtained from all eligible participants before they engage in any study-related activities. Ethics boards will approve any protocol modifications before any changes are implemented.

Study findings will be presented at national and international conferences, in peer-reviewed journals, and via social media posts. A detailed dissemination plan will be developed in collaboration with the local Youth Community Advisory Board in Johannesburg.

## Supporting information

S1 File SPIRIT 2013 checklist. (DOC)
